# What learning theories can teach us in designing neurofeedback treatments

**DOI:** 10.3389/fnhum.2014.00894

**Published:** 2014-11-06

**Authors:** Ute Strehl

**Affiliations:** Institute of Medical Psychology and Behavioral Neurobiology, University of TuebingenTuebingen, Germany

**Keywords:** neurofeedback, learning theories, psychotherapy

## Abstract

Popular definitions of neurofeedback point out that neurofeedback is a process of operant conditioning which leads to self-regulation of brain activity. Self-regulation of brain activity is considered to be a skill. The aim of this paper is to clarify that not only operant conditioning plays a role in the acquisition of this skill. In order to design the learning process additional references have to be derived from classical conditioning, two-process-theory and in particular from skill learning and research into motivational aspects. The impact of learning by trial and error, cueing of behavior, feedback, reinforcement, and knowledge of results as well as transfer of self-regulation skills into everyday life will be analyzed in this paper. In addition to these learning theory basics this paper tries to summarize the knowledge about acquisition of self-regulation from neurofeedback studies with a main emphasis on clinical populations. As a conclusion it is hypothesized that learning to self-regulate has to be offered in a psychotherapeutic, i.e., behavior therapy framework.

## Introduction

“Monkeys meditate for marshmallows”—this is the headline of a report published in the NewScientist in September 2011^1^. Philippens and Vanwersch (2010) had trained marmorset monkeys to increase the sensorimotor rhythm (SMR) of their brains. This was the first time after the famous study of Wyrwicka and Sterman (1968) that it was shown that (even) animals are able to learn self-regulation of brain activity. The very latest neurofeedback studies with animals by Schafer and Moore (2011) and Koralek et al. (2012) will be mentioned below. In humans operant conditioning of electrophysiological brain activity was demonstrated by Kamiya (1966, 2011) who successfully taught subjects to increase and decrease the amount of alpha activity. The success of these trials is attributed to the principles of operant conditioning.

If we consider self-regulation as a skill as it is acknowledged in the brain-computer-interface (BCI) research (Lotte et al., 2013) not only operant conditioning but also classical conditioning, the 2 Process-Theory (Lacroix and Gowen, 1981; Lacroix, 1986) and motivational factors have to be taken into account. Table 1 depicts these paradigms, the involved mechanisms and important variables in skill learning. Section “Basics from Learning Theories to be Considered in Designing a Neurofeedback Protocol” refers to the important variables derived from learning theories and their use in designing neurofeedback protocols. If available, corresponding results from neurofeedback studies with clinical populations will be reported. The paper ends with a short glance at studies regarding the neuronal basis and the therapeutic framework of neurofeedback.

**Table 1:** **Factors involved in acquisition of a skill (after Neumann, 2001, extended and modified)**.

Paradigms	Mechanisms	Important variables
Operant conditioning:	Trial and error	Reinforcement, reinforcer, shaping
**Learning the outcome of a certain behavior**		
Classical conditioning:	Target behavior is associated with (elicited by) conditioned stimuli	Transfer
**Learning to predict important events**		
2 Process theory	1. Phase: operant conditioning of adequate behavior. 2. Phase: association of feedback with interoceptive stimuli.	Instruction; strategies Knowledge of results; feedback; practice
Motivation	Intrinsic / extrinsic	individual differences

While the focus of this hypothesis and theory paper is on theoretical basics and clinical studies it has to be pointed to the growing amount of EEG-neurofeedback studies for optimizing performance. Three consecutive reviews report on the cognitive and affective outcomes in healthy adults (Gruzelier, 2014a), creativity (Gruzelier, 2014b) and on methodological and theoretical considerations (Gruzelier, 2014c).

## Basics from learning theories to be considered in designing a neurofeedback protocol

This section will examine the “mechanisms” and “important variables” as depicted in Table 1 and describe their impact on the design of learning processes with the aim of self-regulation of physiological variables.

### Feedback, reinforcement and knowledge of results

Biofeedback in general is based on real-time feedback of voluntarily induced changes of certain physiological signals. A successful change according to the task is positively reinforced, while failure to change is punished. These basic operant conditioning aspects of biofeedback are harder to access in neurofeedback as there are no receptors to perceive the electrophysiological activity of the brain. The state of the brain can at best be reconstructed by cognitions and emotions. Therefore external feedback is indispensable and it is worth questioning whether self-regulation of brain activity will ever work without any external feedback. The answer can be derived from the animal studies mentioned above. One monkey who increased his SMR activity within 4 sessions of 30 min duration did not receive the reward in the last session. The video shows that he is expecting (and then missing) the marshmallow. He has developed an association between a certain brain activity and its consequences (Philippens and Vanwersch, 2010). Koralek et al. (2012) trained rodents to increase or decrease the pitch of an auditory cursor by 2 different cell assemblies in the primary motor cortex (M1) to either approach sugared water or a food pellet. The tone delivered a continuous feedback of brain activity, which could be rewarded either by food or by sugared water. The rate of correct responses decreased significantly when the feedback was not contingent or when the animals had had free access to the reinforcers before a training session. It was concluded that those “neuroprosthetic skills” were intentionally acquired and goal oriented. The animals changed their behavior (i.e., the activity of certain, distinct cell assemblies) dependent on the reinforcement value of its consequences. The importance of the reinforcement is underlined by Siniatchkin et al. (2000). Children were trained to regulate slow cortical potentials (SCP) and received online wrong feedback but were verbally praised if the trial was successful. From this study as well as from the animal study by Koralek et al. (2012) it may be concluded that positive reinforcement is more important than the operant component of the feedback given!

Contrary to this is what we can derive from the studies regarding the “knowledge of results” by Trowbridge and Cason (1932). Participants were asked to estimate the length of lines. The best results were obtained if quantitative feedback was given, followed by qualitative (“good”, “bad”) feedback. No feedback or senseless syllables yielded the worst results. The best solution is probably the combination of correct feedback and reinforcement. To prevent misunderstandings, the terms “feedback” and “reinforcement” should be differentiated from each other, knowing that feedback is reinforcing in itself. As a consequence a neurofeedback protocol should deliver continuous feedback as regards to the brain activity in question and a positive reinforcement in addition. By scheduling a feedback trial and the subsequent reinforcement the so-called “post-reinforcement synchronization” (PRS) has to be considered. PRS refers to a synchronization of the EEG that was observed in animals (see Sherlin et al., 2011) and in humans (Hallschmid et al., 2002) and is positively correlated with the outcome of learning. With regards to this mechanism, a neurofeedback training session should be discontinuous with many little breaks allowing PRS to take place. According to this, videos or games as feedback seem to be rather unfavorable.

A couple of basic studies, mostly for SCP-Feedback, tried to assess which modality (visual, auditory) and which timing promote learning and whether proportional or binary feedback are preferred. The evidence is rather clear: visual feedback is superior to auditory feedback (Kisil, 1992; Hinterberger et al., 2004), proportional feedback is superior to binary (Travis et al., 1974; Kisil, 1992) and feedback should be as immediate as possible (Kisil and Birbaumer, 1992).

Any of the abovementioned aspects refer not only to the development of the desired behavior but also for undesired behavior. If the system picks up and feeds back behaviors like breathing, eye movements or muscle activity then the patient will learn to demonstrate those behaviors! The outcome is even worse if non-physiological artifacts are fed back. In this case the patient will learn nothing at all because he cannot influence a signal produced e.g., by a faulty electrode. Even worse he will experience loss of control and may develop feelings and cognitions of learned helplessness. As a consequence a proper online artifact-control is mandatory for any equipment.

### Shaping and the question of threshold regulation

Shaping in the operant conditioning paradigm refers to the successive approximation to a new behavior, especially skills. Although claimed by authors the automatic threshold regulation in feedback protocols does not correspond to the prerequisites of a shaping process. Automatic threshold regulation was introduced to make sure that a patient is rewarded at least in 60% or 70% of trials (e.g., Lansbergen et al., 2011). By this in a session a performance may be rewarded that is worse than the performance the day before. From a patient’s perspective whatever he does, in 70% of the time or trials he gets positive reinforcement for sure, even if he is doing nothing. In order to establish a shaping process the final goals as well as the breakpoints on the way to the goal have to be defined. Finally a prognosis whether the achievement of the final goal will be enduring and generalize to similar albeit different situations in life. As an example learning to swim will be broken down in steps like overcoming any water fears, being able to imitate certain movements with help, being able to swim in deep water, being able to swim in deep water for at least 10 min…After this goal is reached parents may allow children to swim without their surveillance expecting that the child will not drown. Neither breakpoints nor final goals nor a prognosis are known for neurofeedback. At best norms are available regarding the amount of activity in a certain frequency band as it is assumed for the theta/beta ratio (Montgomery et al. in Demos 1998) but they were assessed during spontaneous EEG measurements, which cannot be simply transferred to brain activity in a (probably demanding) neurofeedback session. Finally, to date there is no knowledge available regarding change in amplitudes during the treatment and its relation to symptom change. Studies that have found a correlation of performance during so called transfer trials will be mentioned in the next section.

The argument brought forward against automatic threshold regulation partly holds for manual and/or individual regulation of thresholds, too. Whenever the participant “earns” too much or not enough reward, the threshold is adjusted, i.e., is set higher or lower. Again, the idea is to guarantee a certain amount of reward to keep the participant motivated. The motivation becomes more important than the quality of the performance. This is a questionable strategy which should be taken into consideration in future studies.

A few cognitive neuroscience studies with healthy subjects have shown a positive correlation with the amount of changes in amplitudes and cognitive performance (for a review see Gruzelier, 2014a). The open question then is the reference for determining “improvement”: pre-training baseline, first session, last session, pre-session baseline…? The nature of the brain signal that is feedback should make a difference. More stationary activity as in the frequency bands may not need a continuous update of the baseline whereas because of their phasic nature SCP should be continuously (i.e., after each trial) updated. Here it might be more adequate to reinforce any change compared to baseline (see Sherlin et al., 2011).

Again only a few studies have assessed the nature of learning in EEG-feedback (for a review see Gruzelier, 2014c). More knowledge as regards to this issue will help to deal with the question of shaping and threshold regulation.

### Transfer

The transfer of a skill from the setting in which it was acquired to any situation in life where the skill is needed is an important issue. Delivering feedback after every trial compared to intermittent feedback leads to the fastest learning success in motor learning. In the long run however retaining feedback is more successful. Following this observation, Winstein and Schmidt (1990, cited after Mazur, 2002) developed the “guidance hypothesis”. From the very beginning of training, participants experience the same situation they will be confronted with after the training has ended. In addition, they assume that withholding feedback elicits more efforts in memorizing and supports intrinsic motivation.

In 1901, Thorndike and Woodworth put forward the theory of “context specifity” (Thorndike and Woodworth, 1901). They proposed that the degree to which skills transfer to novel situations depends on the number of elements that are identical between the learning context and the novel situation. From animal research, Cartoni et al. (2013) derived the “Pavlovian-Instrumental Transfer Hypothesis” (PIT). They conclude that a conditioned stimulus that is associated to a reward can affect the operant conditioned behavior in different ways. Firstly, an action directed to a goal needs the right context. This is given, if there are cues (in the novel situation) that indicate (from the old context) higher chances to get a reward. This can be realized if the cues from the lab or practitioner’s office are transferred to the everyday life environment (e.g., classroom) and vice versa if cues from everyday life are transferred to the environment where the training takes place. Secondly, an action may have more or less chances to achieve a goal. Successful actions outside the training environment should be rewarded. Finally, the utility of a behavior in a certain situation is evaluated. According to this, cues should help to discriminate situations in which a certain behavior would be useful or not. An increase of theta e.g., might be useful in order to fall asleep while a decrease might be useful to be concentrated and awake. Cue dependent learning constricts learning to the learning environment as long as the cues are not transferred to everyday life situations. The same holds not only for the newly acquired behavior but also for the old, dysfunctional behaviors. Unfavorable stimulus (classroom)—response (inattentive brain states)—reinforcement (punishment, bad marks) associations have to be changed to favorable ones (classroom-attentive brain states-praise, good marks) by associating cues from the classroom with the acquired behavior. This can be done e.g., by simulation class room situations during the training and by bringing cues from the lab to the classroom. Of course the best place for neurofeedback exercises is the classroom—therefore the development of neurofeedback equipment that can be reliably, validly and safely used in real life situations would help a lot!

If the skill is to be transferred with the help of cues these have to be known. A systematic and thorough behavior analysis will help the patient as well the therapist to become aware of eliciting antecedents, be they environmental, emotional, cognitive, behavioral or physiological variables.

Children with Attention Deficit-/Hyperactivity Disorder (ADHD) may have additional problems transferring newly acquired behavior to everyday life. According to Abikoff (2009) this may be caused by the shortened delay gradient and the inability to anticipate consequences, which in turn affect the perception of cues. Being less able to generalize and to discriminate may compromise the transfer. There are two ways out of this dilemma: if neurofeedback is offered in a cognitive behavior therapy context the therapist can take care of disease specific issues (see Disease), and if the self-regulation is automatized and elicited independently of voluntary action (see next section).

### Automation: practice makes perfect

If the self-regulation of brain activity is regarded as a skill automating should be expected as the final aim. The skill is stored in the implicit memory and can be unconsciously retrieved. According to Fitts (1964, cited after Fitts and Posner, 1967) motor learning takes place in three consecutive steps. The “cognitive phase” at the beginning of the process demands a high amount of attentiveness while basis sequences are being learned. The learner will identify by trial and error the correct behavior. During the subsequent “associative” phase the new behavior is practiced, wrong reactions are inhibited if possible. In the end the performance is executed reliably and less attentiveness is needed in this “autonomous, automatic phase”.

The “Two-Process-Theory” (see Table 1) of the acquisition of autonomic control substantiates Fitts’ model. During the cognitive phase the participant tries to identify strategies (see next section) that lead to successful behavior. Thereafter the repeated matching of a reaction and feedback that signals success interoceptive stimuli form an image of a correct reaction, just as shown in the above mentioned monkey meditating for marshmallows. According to Lacroix (1981) biofeedback training leads to autonomic control through a process primarily consisting of the identification of efferent behavioral programs already within the subjects’ repertoire.

In patients with epilepsy who took part in a neurofeedback training of SCP Kotchoubey et al. (2002) showed that self-perception of self-regulation performance developed after patients were successfully able to self-regulate SCPs. Patients who failed in developing self-regulation skills could not correctly estimate their performance. As already mentioned, unlike peripheral motor behavior, electrophysiological activity of the brain is not perceivable. This leads to the question what are the interoceptive stimuli that had been associated with the feedback? Kotchoubey et al. (2002) suggest that changes in the cerebral blood flow, i.e., the extension of receptors of the arterial walls during cortical activation and deactivation might be responsible. Results of a functional imaging study proved an increase of blood flow in different areas of the cortex, depending on the task which was either to produce electrically negative or positive slow potential shifts (Hinterberger et al., 2003). Kotchoubey et al. (2002) provide an alternative explanation by referring to the general control theory. According to this theory subjects perceive operations that are connected with successful control of the cursor (i.e., feedback object) and by this may develop percepts.

Results from long-term studies support the model of skill learning for neurofeedback treatments. After neurofeedback of slow cortical potential patients with ADHD are still able to self-regulate the brain activity 6 months (Leins et al., 2007) and 2 years (Gani et al., 2008) after the end of treatment. In patients with epilepsy successful self-regulation was observed after 1 year (Kotchoubey et al., 2001) and 9 years (Strehl et al., 2014) after the end of treatment. As the activity during activation is more and more concentrated in the area below the sensor this is seen as a further proof for the automation of the skill (Neumann, 2001).

#### Strategies and instructions

If the self-control is achieved through perception of operations as may be derived from the above mentioned control theory the use of a certain strategy seems to be a possible operation. These strategies are rather easy to choose in the case of feedback of spontaneous EEG activity. The correlation between activation and arousal seem to allow easy access to a certain strategy. Very often participants in a theta-/beta-feedback training are instructed to be relaxed and attentive but there is no systematic data available. The use of strategies for SCPs feedback was investigated by Roberts et al. (1989). It was concluded that there are no valid interindividual strategies known. In SCP feedback participants are asked to self-regulate thresholds of cortical excitation. The slow negative potential shift resembles the contingent negative variation (CNV) which can be observed e.g., in a Go/No go experiment. Here the negative shift is provoked by a warning stimulus in expectation of an imperative stimulus after which the subject has to execute a motor reaction as quickly as possible. Therefore Roberts et al. (1989) expected that the imagination of movement preparation would work as a strategy. This was not confirmed by their study; instead strategies differed from subject to subject and even within the subject in the course of the experiment. Today it is thought that the analogy to the CNV does not work because during the feedback trials no imperative stimulus is given. It was concluded that a strategy is the individually developed percept during the associative phase of successful SCP-regulation Neumann (2001). As a consequence it is recommended not to indicate strategies at all. In a study with healthy participants who had to self-regulate the SMR those subjects who reported to use no specific strategy improved best (Kober et al., 2013). Better within session learning of lateralized SCP regulation was observed in a group with healthy participants who did not receive guidance compared to the group who was told to use emotional strategies. A group by session by block by trial analysis showed no performance differences between the groups (Hardman et al., 1997). The authors assume that vivid strategies might overload cognitive resources and that not being able to name the strategy may indicate a more automatic regulation.

#### Practice schedules, how much practice and skill decay?

The seemingly simple question regarding the number of sessions comprises several aspects. How many sessions are necessary until the skill is acquired? How many sessions are needed until reduction of symptoms will be observed? How many sessions will be paid (if at all) by the health insurances? Closely connected is the question regarding the training schedule.

Following the theory of reactive inhibition of Hull (1943) spaced practice yields better retention than massed practice. Accordingly Wang et al. (2014) reported significant improvement after 20 sessions spaced working memory training in healthy children compared to massed training (20 sessions in 2, 5 or 10 days). In the absence of any systematic research on these issues for neurofeedback trainings the bridging from basic theory to encompass cognitive training research and neurofeedback protocols is not easy. Considering that neurofeedback sessions normally last 20 to 60 min more than one session per day does not seem to be possible simply for practical reasons. There are two more aspects to consider. Firstly, how big does the interval between 2 sessions has to be in order to declare a training to be spaced—e.g., 1 day or 1 week? Secondly, does it make a difference which system is being trained—cognition, contingencies between behavior and its reinforcement, or a physiological parameter? Arnold et al. (2013) observed no difference in outcome and parents’ satisfaction after two vs. three weekly sessions, although parents preferred the schedule with three sessions a week.

The question regarding the number of sessions refers to the prognosis of learning success. Basically a positive correlation between successful self-regulation and clinical outcome is subsumed. From clinical practice it is well-known that the picture is more complicated. One patient may have succeeded in self-regulation without improving clinically, the other may have improved clinically without being able to self-regulate in a reliable manner, or self-regulation and outcome may correlate. From research it is only known that in neurofeedback protocols including transfer trials where no feedback is given, the performance during transfer trial predicts the clinical outcome (Strehl et al., 2005 for epilepsy patients; Strehl et al., 2006; Drechsler et al., 2007 for children with ADHD). These studies used SCP-protocols. Drechsler et al. (2007) as well as Strehl et al. (2005) used a significant differentiation between tasks (cortical negativation and cortical positivation) as a marker for learning success, while Strehl et al. (2006) chose a significant negative shift from baseline as criterion. A significant correlation between the number of sessions and decrease of symptoms of inattention was reported as a result of a meta-analysis by Arns et al. (2009). The relation between number of sessions and schedule has not been investigated so far.

Blume (2012) showed in her thesis that different studies used diverse criteria such as significant shifts of potentials compared to baseline, a differentiation between parameters (if more than one was trained), number of correct shifts, and duration of the correct shifts as well as a ratio between certain criteria. If “correct” shifts are to be chosen again criteria are needed. Finally it has to be decided which time points should be included as the amount of data produced in any of N sessions is huge. In her analysis of SCP-FB sessions she decided to classify participants as a “learner” if an a- priori defined differentiation and a reliable negativation was shown in the last session. As a result at the follow-up session 6 months after the end of training some children could now be labeled as a “learner” who were previously classified as a “non-learner”. It was concluded that the learning is ongoing and learning success cannot be predicted from the performance during the sessions. If these results can be replicated and proven to be clinically valid other criteria have to be developed in order to allow an early prognosis. For a small sample of children with ADHD it was demonstrated that the reduction of symptoms of inattention correlated positively with the change of mean amplitudes of negative shifts during training session 5 and 9, but not in session 13, due to an increase in negativities in children with poor outcome (Gevensleben et al., 2014). The authors hypothesize that these children might have needed a prolonged training. Searching for predictors within the training performance would help to individually tailor the treatment.

According to Singer (1980) learning curves in motor learning show that the task, its difficulty, duration and number of repetitions as well as individual variables influence the learning progress. The impact of individual variables is demonstrated in the so-called “overtraining” (Kreider et al., 1998, cited after Blume, 2012). The extent to which practice can lead to further improvement decreases with the extent of practice. Too many sessions may be disadvantageous if a participant is a quick learner. Blume (2012) observed participants who fulfilled the criteria as “learner” rather early after 12 sessions and fell off in quality after the second training phase containing 13 more sessions. In the follow-up evaluation they showed a good performance again. It is concluded that speed of learning differs in individuals possibly according to age, maturation of brain, stress vulnerability and / or cortical functioning (see below—individual factors).

Although skills can last a lifetime they do deteriorate with non-use. As mentioned above according to follow-up studies after SCP-feedback in epilepsy and ADHD, patients not only continued to improve clinically after the end of treatment, self-regulation of brain activity was improved or sustained. It may be concluded that learning does not stop with the last session. By using the self-regulation skill in everyday life patients are being reinforced to be less hyperactive, impulsive and inattentive. This in turn consolidates the behavior while unfavorable brain activity is being extinguished. The skill is used automatically whenever it is needed—in the end it can be assumed that the functioning of the brain has changed.

### Individual variables

The acquisition of the skill to self-regulate brain activity is not only based on certain rules or laws of learning. As already mentioned in the last section, individual variables have to be taken into account, too.

#### Motivation

According to Hofmann et al. (2012) participants need sufficient motivation to invest effort to overcome the discrepancy between the gap between actual and potential performance and obstacles and temptations along the way. Achievement motivation as hope for success or fear of failure and attribution styles are individual variables that might influence the intrinsic as well as the extrinsic motivation. There is limited data on the impact of these variables from neurofeedback and BCI-research available. Witte et al. (2013) observed in healthy participants that control beliefs as regards to technology correlated negatively with the ability to self-regulate SMR. It is assumed that a locus of control might lead to emotional or cognitive overload, which negatively influences the performance.

Intrinsic motivation can be spoiled by inflated praise (Brummelman et al., 2014) and may be enhanced by feedback, which draws attention to a skill without making a judgment about the individual or reporting on feelings. As a general rule an inherently interesting or enjoyable task promotes intrinsic motivation. Therefore it is discussed how to offer feedback sessions to be as interesting as possible. The succession of repeated trials seems to be contraindicated. Trainer or therapists sometimes ask for protocols that use different levels of expertise, similar to computer games. This analogy does not work because self-regulation of brain activity does not improve in a linear manner. As in any motor learning it is not the animation that leads to an improved technique. Instead the execution of the correct behavior (guided by the trainer) is reinforcing in itself. For patients with ADHD it is known that they may perform very well if the task is entertaining, however when facing monotonous tasks the symptoms become obvious. As a consequence a boring training would simulate difficult situations in everyday life. On the other hand loss of motivation has to be avoided—a difficult tightrope walk. Again the therapist is responsible in guiding the treatment and helping the patient to overcome frustration and moments of boredom.

#### Cognition

Results on the impact of cognitive variables as memory or attention are not consistent (Daum et al., 1993; Holzapfel, 1998). Intelligence did not turn out to be a prerequisite of successful self-regulation in Holzapfel et al. (1998) who treated a patient with an IQ below 80.

With regards to brain resources, Wangler et al. (2011) showed that in children with ADHD a larger CNV before training predicted a bigger improvement after training.

#### Disease

Factors being correlated with a disease might influence the performance, too. In locked-in patients with Amyothrophic Lateral Sclerosis (ALS) e.g., moods, bodily complaints, and quality of care influence the performance (Neumann, 2001).

For many years it was assumed that patients with an impairment of executive functions would not be able to learn self-regulation. Maintenance and updating of relevant information, inhibition of irrelevant impulses and mental set shifting are features of the working memory which are necessary for self-regulation (Hofmann et al., 2012). Although they are impaired e.g., in patients with ADHD, results from neurofeedback treatments show, that these patients nevertheless successfully complete the training (e.g., Strehl et al., 2006; Mayer et al., 2012).

In some diseases the typical symptoms may prevent taking part in neurofeedback training. For example an autistic child will not allow being touched or to having electrodes fixed on head and face. In this case a well-trained therapist will implement a shaping program in order to establish trust and compliance.

## Neuronal basis of neurofeedback learning

Due to the use of intracranial electrodes, the latest animal studies deliver insight into the neuronal basis of neurofeedback learning. According to Koralek et al. (2012) striatal neurons change their firing rates and build strong connections with motor cortex neurons. If by experimental manipulations these connections cannot develop the animal is not able to learn the skill. The authors conclude that corticostriatal plasticity is the basis not only for abstract skill learning but also for learning intentional neuroprosthetic skills in the absence of movements.

The specificity of neurofeedback was proven by Schafer and Moore (2011). Rhesus monkeys learned to voluntary reduce or enhance the activity of neurons within the frontal eye field. The pitch of a tone was used as feedback and juice was given as reinforcement. This operant conditioned behavior was associated with improved selective visual attention. The authors suggest that the specific association of self-regulated neural activity with top-down attention may constitute a basis for the observed improvements in patients with ADHD after neurofeedback.

## Conclusion: neurofeedback and psychotherapy

Neurofeedback is not a magic box easily delivered to the patient. Neurofeedback as well as biofeedback for patients will always take place within a patient—therapist interaction. “My experience with years of biofeedback training with various physiological modalities leaves me with the conviction that a very large portion of the total influences on learning is bio-social in nature, testifying to the evolution of the species as a social species. Though seldom discussed in the scientific literature, the nature of interpersonal relations between trainer and trainee are often decisive for learning progress.” (Kamiya, unpublished, retrieved in Neumann, 2001, p. 32).

The *equipment is a tool* within this interaction, *neurofeedback is a method* of behavior therapy. As in any other behavior therapy the therapist initiates and helps through a process during which the patient may learn a new behavior that helps to overcome his symptoms. Different from the usual bottom-up targets in behavior therapy, which are overt behavior, cognitions and emotions, neurofeedback tries to directly change cortical activity. But with the help of the equipment brain activity becomes overt, too. The therapist will need to know the laws of learning as well as how to applicate neurofeedback training in order to be a competent partner in this top-down behavior therapy approach.

## Conflict of interest statement

The author declares that the research was conducted in the absence of any commercial or financial relationships that could be construed as a potential conflict of interest.

## Footnotes

^1^http://www.newscientist.com/article/dn20989-monkeys-meditate-for-marshmallows.html
